# 12/15-Lipoxygenase Is an Interleukin-13 and Interferon-*γ* Counterregulated-Mediator of Allergic Airway Inflammation

**DOI:** 10.1155/2010/727305

**Published:** 2010-09-29

**Authors:** Alexa R. Lindley, Margaret Crapster-Pregont, Yanjun Liu, Douglas A. Kuperman

**Affiliations:** Allergy-Immunology Division, Department of Medicine, Feinberg School of Medicine, Northwestern University, Chicago, IL 60611, USA

## Abstract

Interleukin-13 and interferon-*γ* are important effectors of T-helper cells. Interleukin-13 increases expression of the arachidonic acid-metabolizing enzyme, 15-lipoxygenase-1, in a variety of cell types. 15-lipoxygenase-1 is dramatically elevated in the airways of subjects with asthma. Studies in animals indicate that 15-lipoxygenase-1 contributes to the development of allergic airway inflammation but is protective in some other forms of inflammation. We tested the hypothesis that the ability of interleukin-13 and interferon-*γ* to counterregulate allergic airway inflammation was potentially mediated by counterregulation of 12/15-lipoxygenase, the mouse ortholog of 15-lipoxygenase-1. The airways of mice were treated with interleukin-13 or interferon-*γ* one day prior to each of the four allergen exposures. Interleukin-13 augmented and interferon-*γ* inhibited allergic airway inflammation independently of systemic IgE and mucosal IgA responses but in association with counterregulation of 12/15-lipoxygenase. Interleukin-13 and interferon-*γ* counterregulate 12/15-lipoxygenase potentially contributing to the effects of these cytokines on allergic airway inflammation.

## 1. Introduction

The T-helper (Th) type 2 cytokines, interleukin (IL)-4 and IL-13, and the Th1 cytokine, interferon (IFN)-*γ*, are important effectors of allergic immune responses (as reviewed in [1]). IL-4 and IL-13 share the ability to signal via the type 2 IL-4R (as reviewed in [2]) and the levels of IL-4 [3] and IL-13 [4, 5] are increased in the airways of subjects with asthma. Individuals with asthma have the capacity to produce increased levels of IFN-*γ* [6–9]. However in response to allergen, subjects with atopic asthma have reduced IFN-*γ* production as compared to atopic nonasthmatic subjects [10] and patients with unresolved asthma have reduced IFN-*γ* production as compared to subjects with resolved asthma and control subjects [11]. In previous studies, treatment of mouse airways with IL-13 induces eosinophilic airway inflammation [12–14]. IL-13 is known to induce the arachidonic acid metabolizing enzyme 15-lipoxygenase-1 (15-LO-1) in a variety of cultured human cells including blood monocytes [15], normal bronchial epithelial cells [16] and dendritic cells [17]. Further, blockade of IL-4 and IL-13 signaling locally in the airway provides significant protection in clinical studies of asthma [18]. In contrast to the effects of IL-4 and IL-13, inhalation of IFN-*γ* decreases eosinophilic inflammation in the airways of subjects with asthma [19] and treatment of mouse airways with IFN-*γ* inhibits eosinophilic airway inflammation [13, 20]. IFN-*γ* also inhibits the IL-4-induced expression of 15-LO-1 in cultured human monocytes [21]. Thus, the evidence suggests that the balance of IL-13 and IFN-*γ* levels in the airway is important in determining the levels of local 15-LO-1 expression and the severity of airway inflammation in asthma. 

 The 15-LO-1 enzyme and its mouse ortholog, 12/15-LO, insert molecular oxygen at the 12th or 15th carbon of arachidonic acid (AA) resulting in the generation of 12(*S*)- and 15(*S*)-hydroxyeicosatetraenoic acid** (**HETE) [22]. The 15-LO-1 enzyme is normally expressed in airway epithelial cells, eosinophils, mast cells and dendritic cells [23–27]. The overall contribution of the enzyme and its products appear to be organ and disease model dependent. For example, it has a protective role in animal models of ocular epithelial wound healing [28], atherosclerosis [29], arthritis [30], and periodontal inflammation [31]. The protective role of 15-LO-1 is hypothesized to be mediated by the interaction of its products with 5-LO resulting in the generation of potent proresolving mediators including lipoxin A4 (LXA4), resolvin E1, and protectin D1 (as reviewed in [32]). For example, LXA4 treatment blocks allergic pleural eosinophil influx in rats [33] and protectin D1 treatment rescues 15-LO-1 deficient cells from oxidative stress-induced apoptosis [34]. There are some circumstances in which 15-LO-1 contributes to pathology. For example, 12/15-LO overexpressing mice develop spontaneous increases in aortic fatty streak lesions associated with increased monocyte adhesion to aortic endothelial cells. These atherosclerotic changes were potentially explained by increased expression of ICAM on aortic endothelial cells [35]. In cultured adipocytes, 12(*S*)-HETE treatment induces the expression of genes encoding proinflammatory cytokines [36]. In mouse models of allergic asthma, the overall role of 12/15-LO appears to be proinflammatory whether elicited by allergen alone [37, 38] or by the combination of allergen and double-stranded RNA [39]. 

 Clinical studies suggest that 15-LO-1 is important in asthma. As compared to healthy control subjects and subjects with mild to moderate asthma, the expression and activity of 15-LO-1 is dramatically elevated in epithelial cells and inflammatory cells in the airways of individuals with severe asthma [40]. A member of our group previously reported that as compared to healthy control subjects, the levels of 15-LO-1 transcripts are slightly elevated in airway epithelial cells from individuals with mild and stable asthma [41]. As compared to nonsevere asthmatics, individuals with severe asthma had decreases of 15-LO-1 expression in BAL cells and increases of 15-LO-1 in bronchial biopsies with dramatic decreases of LXA4 in BAL fluid [42]. 

 Based on these observations, we sought to determine if IL-13 and IFN-*γ* coordinately counterregulate allergic airway inflammation and 12/15-LO in the airways of mice. We observed augmentation of allergic airway inflammation by IL-13 and inhibition of allergic airway inflammation by IFN-*γ* in the airways of mice. The counterregulatory effects of these cytokines on allergic airway inflammation were not clearly explained by changes in systemic and mucosal immunoglobulin responses or by changes in LXA4 levels. However, the augmentation of allergic airway inflammation by IL-13 was associated with augmentation of 12/15-LO and the inhibition of allergic airway inflammation by IFN-*γ* was associated with inhibition of 12/15-LO. Given that 12/15-LO contributes to the development of allergic airway inflammation in mice [37, 38], the results suggest that the balance of IL-13 and IFN-*γ* levels in the airway might be an important factor that counterregulates 15-LO-1 and as a consequence, the severity of allergic airway inflammation in asthma. 

## 2. Materials and Methods

### 2.1. Mice

The experiments were approved by the Northwestern University Animal Care and Use Committee and complied with the “Guide for the care and use of laboratory animals” published by the National Academy Press (revised 1996). C57Bl/6 female 6-7-week-old mice (Jackson Laboratories, Bar Harbor, Me) were evaluated.

### 2.2. Protocols

Mice were treated with 1.5% chicken-egg ovalbumin (OVA) in 50 *μ*l phosphate-buffered saline (PBS) or an equivalent volume of PBS (controls) via the intratracheal (i.t.) route once every 4 days for a total of four treatments. Mice received 5.0 *μ*g rmIL-13 or 5.0 *μ*g rmIFN-*γ* (PeproTech, Rocky Hill, NJ) in 50 *μ*l PBS or an equivalent volume of PBS (controls) via the i.t. route one day prior to each OVA treatment. Tissue samples were harvested four days following the fourth OVA treatment.

### 2.3. Lung Inflammation

Right lungs were immersed in 10% formalyn under vacuum for 24 hours then serially dehydrated and embedded in paraffin. Sections (5 *μ*m) were stained with hematoxylin and eosin. Sections from 5 mice/group were analyzed by light microscopy for the density of inflammation surrounding airways and representative images are shown. Bronchoalveolar lavage fluid (BALF) was collected via a tracheotomy with a single aliquot of 0.9 ml of PBS. The BALF cells were counted and stained for identification by light microscopy.

### 2.4. Immunoglobulins

Blood was collected into tubes after excision of a kidney and allowed to coagulate at room temperature for 30 minutes. Serum was collected following separation from blood cells by centrifugation. Total immunoglobulin (Ig)E was measured in serum that was diluted 1 : 50 in PBS by means of a specific ELISA kit (R&D systems, Minneapolis, Minn). Ova-specific IgG1 was detected by direct ELISA as described [43]. Reagents were not available for Ova-specific IgG1 standard curves. Therefore, optical density values from 4-fold serially diluted samples were compared to estimate fold differences between groups. Total IgA was measured in cell-free BALF without dilution by means of a specific ELISA kit (Immunology Consultants Laboratories, Inc, Newberg, Ore).

### 2.5. Metabolites

12(*S*)-HETE, 15(*S*)-HETE (Assay Designs, Ann Arbor, Mich) and LXA4 (Cayman Chemical, Ann Arbor, Mich) were measured in cell-free BALF without dilution by means of specific ELISAs.

### 2.6. Secretory Component

Ninety-six well plates were incubated overnight with cell-free BALF without dilution and then 1 : 200 goat antimouse secretory component (SC; R&D systems), followed by 1 : 1000 horseradish peroxidase-conjugated donkey antigoat IgG and TMB substrate (BD Biosciences, San Jose, Calif). Concentrations in samples were determined by means of comparison with optical density values generated by BALF to which known amounts of rmSC (R&D systems) were added. OVA-specific SC was determined by incubating 96-well plates overnight with 0.1% OVA, followed by cell-free BALF diluted 1 : 8, followed by 1 : 200 goat antimouse SC (R&D systems), followed by 1 : 1000 horseradish peroxidase-conjugated donkey antigoat IgG and TMB substrate (BD Biosciences, San Jose, Calif). Due to the lack of purified OVA-specific SIgA to generate a standard curve, optical density values are reported.

### 2.7. Transcripts

The left lungs were homogenized in Trizol (Sigma, St Louis, Mo) and 2.0 *μ*g total RNA was converted to cDNA by means of reverse transcription. Published primer sequences [38] were used to detect GAPDH, IL-4, IL-5, IL-13, IFN-*γ*, pIgR, and 12/15-LO transcripts by means of the Taqman method of real-time PCR. Copy numbers were normalized to that of GAPDH.

### 2.8. Statistics

The Sigma-Stat V.11 software package was used to perform ANOVA using the all pair wise multiple comparisons procedures (Holm-Sidak method). Means and SEMs are shown for 6 mice per group. *P*-values ≤.05 were considered statistically significant.

## 3. Results and Discussion

**Figure 1:**
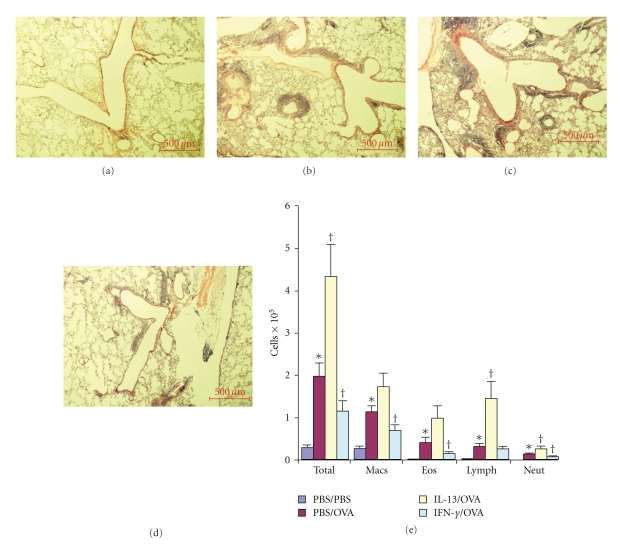
Effects of IL-13 and IFN-*γ* on allergic airway inflammation. Representative images (5x magnification) of lungs showing airway associated tissue inflammation from PBS/PBS (a), PBS/OVA (b), IL-13/OVA (c) and IFN-*γ*/OVA (d) mice. Scale bars are 500 *μ*m. Levels and types of cells in BALF (e) from PBS/PBS, PBS/OVA, IL-13/OVA and IFN-*γ*/OVA groups of mice. *P-value ≤.05 versus PBS/PBS. ^†^ *P*-value ≤.05 versus PBS/OVA.

Allergic control mice were generated by treatment of their airways with PBS one day prior to each of four 1.5% OVA treatments to their airways occurring 4 days apart (PBS/OVA). Nonallergic control mice received PBS one day prior to each of four PBS-treatments occurring 4 days apart (PBS/PBS). These groups of control mice were compared to a group of mice that were treated with 5.0 *μ*g rmIL-13 one day prior to each 1.5% OVA treatment (IL-13/OVA) and another group of mice that were treated with 5.0 *μ*g rmIFN-*γ* one day prior to each 1.5% OVA treatment (IFN-*γ*/OVA). All measurements were made 4 days following the 4th OVA (or PBS; controls) treatment. PBS/PBS mice (Figure 1(a)) had no obvious inflammatory infiltrates in their lungs. PBS/OVA mice (Figure 1(b)) developed modest airway associated inflammatory infiltrates in their lungs. As compared to PBS/OVA mice, IL-13/OVA mice (Figure 1(c)) developed dramatically more airway-associated inflammatory infiltrates. As compared to PBS/OVA mice, IFN-*γ*/OVA mice (Figure 1(d)) developed less airway-associated inflammatory infiltrates. We quantified the lung inflammation by counting the number of macrophages, eosinophils, lymphocytes and neutrophils in BALF (Figure 1(e)). As compared to PBS/PBS mice, PBS/OVA mice developed significant increases of total cells and significant increases of each cell type. As compared to PBS/OVA mice, IL-13/OVA mice developed significant increases of total cells, significant increases of lymphocytes, significant increases of neutrophils, not-quite significant increases of macrophages (*P* = .09) and not-quite significant increases of eosinophils (*P* = .07). As compared to PBS/OVA mice, IFN-*γ*/OVA mice developed significant decreases of total cells, significant decreases of macrophages, significant decreases of eosinophils, no decrease of lymphocytes and significant decreases of neutrophils. Overall, these results indicate that IL-13 in the airway augments the development of allergic airway inflammation in mice and IFN-*γ* in the airway inhibits the development of allergic airway inflammation in mice. 

**Figure 2:**
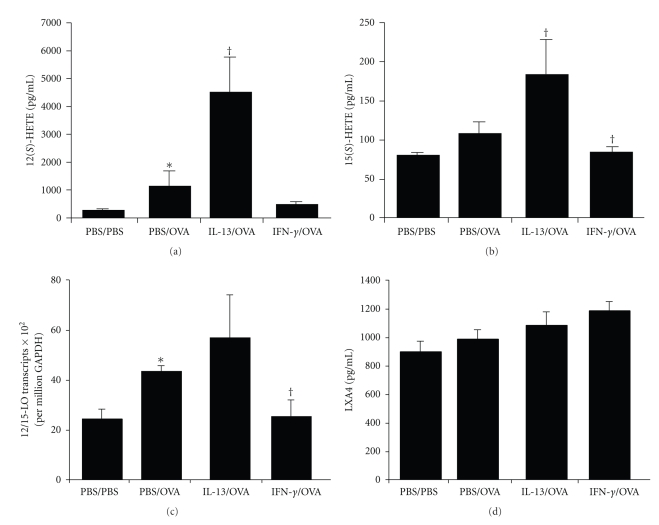
Effects of IL-13 and IFN-*γ* on 12/15-LO. Levels of 12(*S*)-HETE (a) and 15(*S*)-HETE (b) and LXA4 (c) in BALF and 12/15-LO transcripts in whole lung (d) from PBS/PBS, PBS/OVA, IL-13/OVA and IFN-*γ*/OVA groups of mice. *P-value ≤.05 versus PBS/PBS. ^†^ *P*-value ≤.05 versus PBS/OVA.

 To determine the influence of IL-13 and IFN-*γ* in the airway on the 12/15-LO enzyme, we measured the levels of its AA-derived metabolites, 12(*S*)-HETE (Figure 2(a)) and 15(*S*)-HETE (Figure 2(b)) in BALF. As compared to PBS/PBS mice, PBS/OVA mice developed significant increases of 12(*S*)-HETE and no significant increase in 15(*S*)-HETE. As compared to PBS/OVA mice, IL-13/OVA mice developed significant increases of both 12(*S*)-HETE and 15(*S*)-HETE. As compared to PBS/OVA mice, IFN-*γ*/OVA mice did develop significant decreases in 15(*S*)-HETE but did develop a trend toward attenuated 12(*S*)-HETE levels considering that these values were not significantly different from PBS/PBS mice. To help confirm these results, we measured whole lung gene transcript levels for 12/15-LO (Figure 2(c)). As compared to PBS/PBS mice, PBS/OVA mice developed significant increases of 12/15-LO transcript levels. As compared to PBS/OVA mice, IL-13/OVA mice had no increase and IFN-*γ*/OVA mice had significant decreases of 12/15-LO transcript levels. We attempted to detect a possible influence of IL-13 and IFN-*γ* in the airway on LXA4 production (Figure 2(d)). The potent proresolving metabolite LXA4 can be generated as a consequence of an interaction between 12/15-LO metabolites and 5-LO. In spite of LXA4 being subject to rapid degradation, we detected almost significant (*P* = .06) increases of LXA4 in IFN-*γ*/ OVA treated mice as compared to PBS/PBS treated mice. As compared to the PBS/OVA group, there were small trends toward increases but not significant changes of LXA4 levels in either the IL-13/OVA or IFN-*γ*/ OVA groups. The results suggest that IL-13 and IFN-*γ* counterregulate 12/15-LO in the airway. Although small quantities of proresolving mediators can have large biologic effects, we conclude that the resolution of inflammation mediated by IFN-*γ* in this study was not associated with obviously increased levels of LXA4 in BALF.

**Figure 3:**
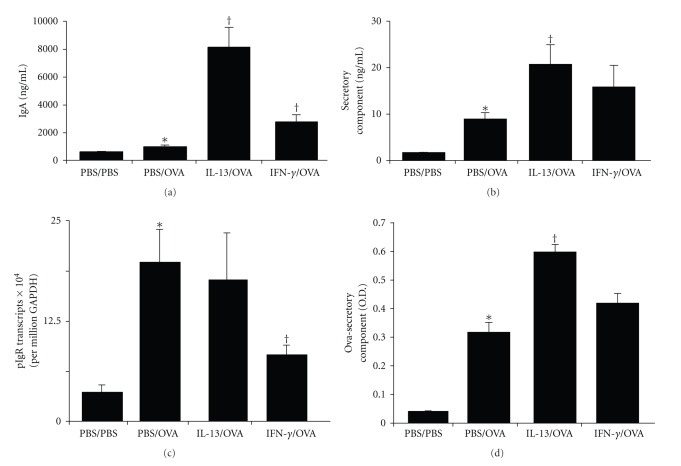
Effects of IL-13 and IFN-*γ* on SIgA. Levels of IgA in BALF (a), SC in BALF (b), whole lung pIgR transcripts (c) and OVA-bound SC in BALF (d) from PBS/PBS, PBS/OVA, IL-13/OVA and IFN-*γ*/OVA groups of mice. *P-value ≤.05 versus PBS/PBS. ^†^ *P*-value ≤.05 versus PBS/OVA.

 As compared to wild-type mice, 12/15-LO knockout mice have increased SIgA in their airways [38]. We investigated the effects of IL-13 and IFN-*γ* in the airway on SIgA levels because it is a potential mediator of the effects of 12/15-LO on allergic airway inflammation. In the process of active epithelial-mediated IgA transport, the extracellular domain of the polymeric immunoglobulin receptor (pIgR), termed secretory component (SC), is cleaved at the apical epithelial surface and remains covalently bound to polymeric (p) IgA resulting in release of SIgA into the lumen (as reviewed in [44]). Therefore, to determine the effect of IL-13 and IFN-*γ* in the airway on SIgA levels, we measured total IgA (Figure 3(a)) and total SC (Figure 3(b)) in BALF. As compared to PBS/PBS mice, PBS/OVA mice developed significant increases of total IgA and significant increases of total SC. As compared to PBS/OVA mice, IL-13/OVA mice developed significant increases of total IgA and significant increases of total SC. As compared to PBS/OVA mice, IFN-*γ*/OVA mice had small but significant increases of total IgA but did not have significant increases of total SC. Together, these results indicate that SIgA levels were increased by IL-13. While total IgA was somewhat increased by IFN-*γ* in the airway, this might not have reflected an increase of SIgA. To help confirm these results, we measured the lung gene transcript levels for pIgR (Figure 3(c)). As compared to PBS/PBS mice, PBS/OVA mice developed significant increases of pIgR transcript levels. As compared to PBS/OVA mice, IL-13/OVA mice did not develop increases of pIgR transcripts. As compared to PBS/OVA mice, IFN-*γ*/OVA mice developed significant decreases of pIgR transcript levels. Together with the total IgA and SC results, these data indicate that SIgA levels were increased by IL-13 in the airway and SIgA levels were not increased by IFN-*γ* in the airway.

 Following repeated OVA exposures to the airway one would expect that the local mucosal adaptive immune response would result in the generation of OVA-specific SIgA. As an indirect measurement of OVA-specific SIgA, we detected SC that remained bound to OVA coated 96-well plates (Figure 3(d)). As compared to PBS/PBS mice, PBS/OVA mice developed significant levels of SC that remained bound to OVA and considering the increase of total IgA and total SC in these mice, this likely reflects a significant increase of OVA-specific SIgA. As compared to PBS/OVA mice, IL-13/OVA mice developed significant increases in SC that remained bound to OVA and considering the increase of total IgA and total SC in these mice, this likely reflects a significant increase of OVA-specific SIgA. As compared to PBS/OVA mice, IFN-*γ*/OVA mice did not have significantly increased SC that remained bound to OVA and therefore likely did not develop increased levels of OVA-specific SIgA. 

**Figure 4:**
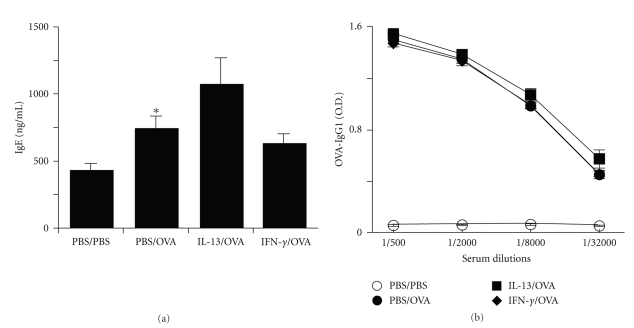
Effects of IL-13 and IFN-*γ* on serum Ig levels. Levels of IgE (a) and OVA-specific IgG1 (b) in serum from PBS/PBS, PBS/OVA, IL-13/OVA and IFN-*γ*/OVA groups of mice. *P-value ≤.05 versus PBS/PBS.

 To determine if the influence of IL-13 and IFN-*γ* in the airway on allergic airway inflammation was possibly due to effects on allergic systemic sensitization, we measured IgE and OVA-specific IgG1 levels in serum (Figure 4). As compared to PBS/PBS mice, PBS/OVA mice developed significant increases of serum IgE and OVA-specific IgG1 levels. Although small trends were present for IgE, IL-13/OVA mice did not develop significant increases of serum IgE or OVA-specific IgG1 levels as compared to PBS/OVA mice and IFN-*γ*/OVA mice did not develop significant decreases of serum IgE or OVA-specific IgG1 levels as compared to PBS/OVA mice. These results suggest that an influence of IL-13 and IFN-*γ* on allergic systemic sensitization is not likely to explain their effects on allergic airway inflammation.

**Figure 5:**
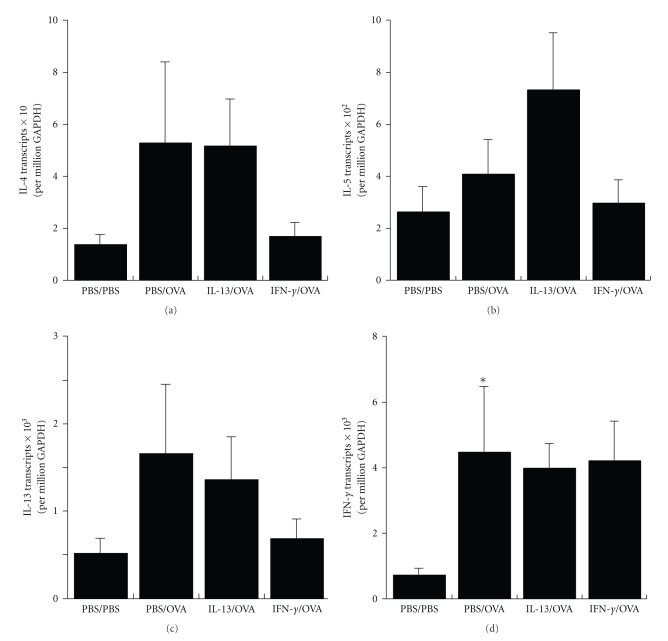
Effects of IL-13 and IFN-*γ* on Th1 and Th2 cytokine transcripts. Levels of IL-4 (a), IL-5 (b), IL-13 (c) and IFN-*γ* (d) whole lung transcripts from PBS/PBS, PBS/OVA, IL-13/OVA and IFN-*γ*/OVA groups of mice. *P-value ≤.05 versus PBS/PBS.

 To evaluate if the influence of IL-13 and IFN-*γ* in the airway on allergic airway inflammation was possibly due to effects on endogenous Th2 and Th1 cytokines, we measured whole lung gene transcript levels for IL-4, IL-5, IL-13, and IFN-*γ* (Figure 5). As compared to PBS/PBS mice, PBS/OVA mice developed small trends but no significant increase of IL-4, IL-5, and IL-13 (Th2 cytokine) transcript levels. However, PBS/OVA mice developed significant increases of IFN-*γ* (Th1 cytokine) transcript levels. Although small trends were present, IL-13/OVA mice did not develop significant increases in cytokine transcripts as compared to PBS/OVA mice and IFN-*γ*/OVA mice not develop significant decreases in cytokine transcripts as compared to PBS/OVA mice. These results suggest that an influence of IL-13 and IFN-*γ* on endogenous Th2 and Th1 cytokines is not likely to explain their effects on allergic airway inflammation.

 In this study we observed that IL-13 in the airway augments and IFN-*γ* in the airway inhibits the severity of allergic airway inflammation. These results are consistent with the results of other similarly designed studies. For example, IL-13 delivered to the airway is sufficient to induce eosinophilic airway inflammation [12–14] and IFN-*γ* delivered to the airway inhibits allergic airway inflammation [13, 20] in mice. Consistent with the proinflammatory role of IL-13 in the airway, blockade of IL-13 exclusively in the airway [14, 45] and IL-13 gene deletion protects mice from allergic airway inflammation [46]. Consistent with the antiinflammatory role of IFN-*γ* in the airway, IFN-*γ* receptor gene deletion augments allergic airway inflammation [47] and systemic treatment of mice with an IFN-*γ* expressing plasmid inhibits allergic airway inflammation [48]. 

 These results indicate that the effects of IL-13 and IFN-*γ* in the airway on allergic sensitization and endogenous Th2 and Th1 cytokines do not account for their effects on allergic airway inflammation. There are a diverse array of mechanisms by which it is thought that IL-13 can augment (as reviewed in [49]) and IFN-*γ* can attenuate (as reviewed in [50]) allergic airway inflammation. To the best of our knowledge, the *in vivo* ability of IL-13 and IFN-*γ* to counterregulate 12/15-LO in the airway has not been previously reported. As compared to wild-type mice, 12/15-LO knockout mice are protected from allergic airway inflammation [37, 38]. Therefore, the local counterregulation of 12/15-LO by IL-13 and IFN-*γ* in the airway, as observed for the first time in this paper, might represent an important mechanism by which these cytokines mediate their opposing effects on the severity of allergic airway inflammation. However, it is possible that the counterregulatory effect of IL-13 and IFN-*γ*  on 12/15-LO transcripts, as observed in this study, is not a cause but rather a consequence of the effects of these cytokines on airway inflammation. This is supported by the observation that inflammatory cells in the airways of asthmatics [40], and human eosinophils [27] and Th2-skewed human mononuclear cells [51] are rich sources of 15-LO-1. In addition, it is possible that the location of expression of human 15-LO-1 might not be equivalent to mouse 12/15-LO (both tissue and cell type) and this might lead to differences in their contribution to IL-13 and IFN-*γ* mediated regulation of allergic airway inflammation.

 As compared to wild-type mice, 12/15-LO knockout mice have elevated levels of total SIgA in their airways [38]. Based on this observation one might predict that induction of 12/15-LO by IL-13 would result in suppression of total SIgA. In this study we observed that IL-13 in the airway increased 12/15-LO metabolites. However, IL-13 in the airway unexpectedly increased total SIgA levels. The IL-13-mediated increase of total SIgA levels could have occurred due to a relatively greater ability of IL-13 to increase SIgA by independent mechanisms as compared to a relatively minor compensatory role mediated by induction of 12/15-LO. It is also possible that while deficiency of 12/15-LO augments SIgA levels [38], induction of 12/15-LO does not have the opposite effect. 12/15-LO overexpressing mice are resistant to the onset of atherosclerosis in association with reduced IL-17 and PGE2 with increased protectin D1 levels [29]. Additional studies of these mice would be helpful to determine what the effect of induction of 12/15-LO by itself is on the levels of SIgA in the airway. 

 In this study, we observed augmentation of allergen-specific SIgA by IL-13 in the context of augmented allergic airway inflammation. The classical view is that specific SIgA mediates immune exclusion at mucosal surfaces (as reviewed in [52]). Therefore one might expect that augmentation of specific SIgA would inhibit allergic airway inflammation. On the other hand, studies of cultured human eosinophils [53–55] and clinical studies of asthmatic subjects [56] suggest that SIgA is a potent inducer of eosinophil degranulation. Therefore, augmentation of SIgA by IL-13 might also be predicted to exacerbate the severity of allergic airway inflammation due to release of proinflammatory mediators from eosinophils. However, mouse eosinophils are more resistant to degranulation as compared to human eosinophils [57] and mouse cells do not express the same repertoire of IgA receptors as compared to human cells [58]. Therefore, it is not clear what effect specific SIgA has, if any, on allergic airway inflammation and this might be, at least in part, due to some limitations of mouse modeling.

## 4. Conclusions

The balance of IL-13 and IFN-*γ* in the airway is strongly implicated by clinical and animal studies as an important factor that regulates the severity of allergic airway inflammation. As compared to normal subjects and subjects with mild to moderate asthma, 15-LO-1 is present at dramatically elevated levels in the airways of individuals with severe asthma [40]. In the present study, the counterregulation of allergic airway inflammation by IL-13 and IFN-*γ* in the airway occurred in association with the counterregulation of 12/15-LO. The protection of 12/15-LO-deficient mice from allergic airway inflammation suggests that 15-LO-1 is an important pathologic mediator of airway inflammation in asthma [37, 38]. Although there are likely important differences between mouse 12/15-LO and human 15-LO-1 in the context of different inflammatory settings and in their ability to mediate the production of proresolving metabolites, this study suggests that the counterregulation of 15-LO-1 by IL-13 and IFN-*γ* may represent an important mechanism by which IL-13 and IFN-*γ* mediate their opposing effects on the severity of airway inflammation in asthma.
